# The ratio of nurse consultation and physician efficiency index of senior rheumatologists is significantly higher than junior physicians in rheumatology residency training: A new efficiency measure in a cohort, exploratory study

**DOI:** 10.1097/MD.0000000000006601

**Published:** 2017-04-07

**Authors:** Amir Emamifar, Morten Hai van Bui Hansen, Inger Marie Jensen Hansen

**Affiliations:** a Department of Rheumatology, Odense University Hospital, Svendborg Hospital, Svendborg; b Department of Economy, Aarhus University, Aarhus; c University of Southern Denmark, Odense; d Danbio, Copenhagen, Denmark.

**Keywords:** DAS28, healthcare costs, nurse consultation, physician efficiency, rheumatoid arthritis

## Abstract

To elucidate the difference between ratios of nurse consultation sought by senior rheumatologists and junior physicians in rheumatology residency training, and also to evaluate physician efficiency index respecting patients with rheumatoid arthritis (RA).

Data regarding outpatient visits for RA patients between November 2013 and 2015 were extracted. The mean interval (day) between consultations, the nurse/physician visits ratio, and physician efficiency index (nurse/physician visits ratio × mean interval) for each senior and junior physicians were calculated. Disease Activity Score in 28 joints-C-Reactive Protein (DAS28-CRP) and Health Assessment Questionnaire (HAQ) scores were used to monitor treatment outcome. Therefore, DAS28 and HAQ scores were measured 3 times: firstly at physician consultation, then after nurse consultation, and finally at the third visit, either at a nurse or physician consultation.

Of 6046 visits, 3699 visits, planned by 11 physicians (4 specialists and 7 junior physicians), were included. These numbers of visits belonged to 672 RA patients, among which 431 (64.1%) patients were female, the mean age being 64.9 ± 14.1 years, and DAS28 at baseline was 4.5 ± 1.2. The nurse/physician visits ratio (*P* = .01) and mean efficiency index (*P* = .04) of senior rheumatologists were significantly higher than that of junior physicians. Regression analysis showed a positive correlation between physician postgraduate experience and physician efficiency index adjusted for DAS28 at baseline and number of patients for each physician (regression coefficient 5.427, 95% confidence interval 1.068–9.787, *P* = .022). There was a high correlation between physicians’ postgraduate experience (year) and the ratio of nurse/physician visits (*r* = 0.91, *P* < .001), and also physician efficiency index (*r* = 0.94, *P* < .001). Nurse consultation did not contribute to worsening treatment outcome, since DAS28 and HAQ scores were significantly decreased if physician visits were followed by nurse visits (*P* = .004 for DAS28 and *P* = .025 for HAQ).

If junior physicians are supervised to refer RA patients with milder and sufficient treatment plan to nurses, the entire department operates more efficiently, leading to prevent additional expenses (due to the differences in yearly salary of physicians and nurses) and human resource waste. Quality of care should be monitored by markers of disease activity and CRP.

## 1 Introduction

Due to increasing healthcare system costs, many efforts have been made to improve the effectiveness of the system. The main aim of these is to decrease the medical expenses, with maintaining the quality of care as high as possible. Achieving this goal requires indices to be measured and efficiency of healthcare systems to be compared. Therefore, various measures have been developed to assess efficiency of healthcare providers, and also healthcare systems. Until now, these measures can be categorized into 2 main groups: firstly, measures assess the efficiency of hospitals, and secondly, measures assess the efficiency of personnel including physicians, nurses, and so on. Other measures to evaluate the health plan, and so on, are discussed in the literature less commonly.^[1–5]^ Efficiency has been defined by the Institute of Medicine as “Avoiding waste, including waste of equipment, supplies, ideas, and energy.”^[6]^

Nurse consultation is a vital component of daily practice, and previous research illustrated the positive impact of nurse staffing on patient outcomes.^[7]^ With respect to developing a more efficient healthcare system and preventing waste of medical resources, nurse contribution to daily practice plays an important role to reduce the total expenses of healthcare system, together with providing high-quality care.^[8,9]^ However, we suppose there is a tendency that junior physicians are reluctant to seek nurse consultation sufficiently, which could be a source of economical, due to differences in salaries, and human resource waste. Our primary hypothesis was that the ratio of nurse consultation was higher with senior rheumatologists compared with junior physicians in rheumatology residency training. Furthermore, we hypothesized that the quality of treatment after nurse consultation would remain fine, that is, stable/lower disease activity and functional disability, when well-characterized patients with sufficient treatment plan were referred to a nurse for about every second visit based on physician's individual assessment.

Disease Activity Score in 28 joints-C-Reactive Protein (DAS28-CRP) is a composite score to evaluate disease activity, and also treatment response of patients with rheumatoid arthritis (RA). It is derived from 4 components including: tender joints (TJ) (range 0–28), swollen joints (SJ) (range 0–28), patient global assessment (PGA) (range 0–100), and laboratory values of CRP. It is continuous and ranges from 0.96 to 9.4 (the latter if CRP is 100 mg/L). A DAS28 of >5.1 indicates high disease activity, 3.2 < DAS28 ≤ 5.1 moderate disease activity, and DAS28 ≤3.2 low disease activity.^[10,11]^ At our department, patients with milder diagnosed RA disease are usually referred to junior physicians.

The Health Assessment Questionnaire (HAQ) is a reliable and valid instrument deliberately designed to assess health outcome in multiple chronic illnesses prospectively. The HAQ score illustrates the extent of individual's functional ability and evaluates the usual ability of patients to perform daily tasks over the past week. The HAQ score ranges between 0 and 3, with scores of 0 to 1 representing mild to moderate difficulty, 1 to 2 moderate to severe disability, and 2 to 3 severe to very severe disability. The average score of 1.2 for RA has been reported compared with average score of 0.49 in population-based study.^[12]^

We performed a study to delineate the interval between consultations, together with ratio of nurse consultation for each senior rheumatologist compared with junior physicians in rheumatology residency training, which was a guide to find out how often nurse consultations were sought by senior and junior physicians. To get a more precise evaluation of the physician efficiency, we investigated the intervals between consultations, together with his/her tendency to refer patients to the nurses at succeeding visits for each individual physician, why we introduced the concept of physician efficiency index. Subsequently, the correlation between ratio of nurse consultation, and also physician efficiency index and physicians’ postgraduate experience, was evaluated, because it seemed that physician experience plays a significant role in increasing physician efficiency and, therefore, decreasing healthcare costs.^[13–15]^ DAS28 and HAQ scores were used to monitor the outcome of treatment for patients who had consulted with nurses. Thus, we calculated DAS28 and HAQ scores, using Danbio, 3 times, and compared the results afterwards.

## 2 Methods

### 2.1 Danbio

Danbio registry was established in 2000 and provides nationwide data on the disease characteristic of patients with inflammatory rheumatic disease including RA (eg, diagnosis, diseases duration, treatment, functional status, and disease activity scores). In Denmark, all patients with diagnosis of RA should be registered in Danbio. At our department, all RA patients are registered at every consultation. Each rheumatology department has access to its own patients’ data. Danbio has been approved by The Danish Data Registry (j. nr. 2007–58–0014 and j.nr. 2007–58–0006), and National Board of Health (j. nr. 7–201–03–12/1), and is fully described by Hetland.^[16]^

### 2.2 Study design and setting

This is an observational, exploratory, single-center cohort study. The complete study was performed at the rheumatology outpatient clinic in March 2016. Local ethical approval was sought from Danish Data Protection Agency (file no. 16/8974).

### 2.3 Specialist nursing roles

The role of registered nurse in the management of rheumatic diseases differs remarkably across countries and regions by the reason of the level of education, trainings, and expertise, and also national and regional regulations. The specialist nurses, who have trained in the field of rheumatology, may not exist in some countries; however, this has developed in other countries, where specialist nurses are responsible to perform different procedures including self-management support, patient education, intra-articular injections, recommendation/dose adjustment of some drugs, monitoring of disease activity, monitoring of treatment (eg, disease-modifying and biological drugs), telephone consultation, and hospital admission.^[17–19]^ A 10-item recommendation for the role of the nurses in the management of chronic inflammatory arthritis has been published by The European League Against Rheumatism (EULAR) using a combination of evidence-based and expert consensus.^[19]^ The role of specialist nurses in our clinic is also in line with EULAR recommendation; however, there might be few differences due to the national/regional regulations. In our clinic, nurses undertake continuous education and training to improve and maintain their knowledge with respect to the management of rheumatic diseases. They are responsible for disease monitoring (eg, disease activity assessment, joint examination, etc), treatment monitoring (steroid treatment, disease modifying, biologics), carrying out interventions (intra-articular injections, diagnostic procedures, etc), and addressing physical, psychological, and social problems. Moreover, they provide education to the patients to improve their knowledge throughout the course of the disease at every consultation. The average years of experience of nurses working at the clinic were about 10 years.

### 2.4 RA patient referral

Patients diagnosed with RA need long-term follow-up. In our outpatient clinic, RA patients are firstly seen by physicians—either senior rheumatologist or junior physicians in rheumatology residency training—who perform initial clinical evaluation and order blood tests and imaging evaluation. Patients will generally be consulted with the same physician in the next visit, when the results of blood tests and imaging are ready, and subsequently appropriate treatment will be started. Thereafter, the patients will be seen by physicians or nurses in the following visits. This is the physician decision that patients should refer to the nurses for follow-up, when the diagnosis of RA is made and the course of disease seems stable, or perhaps should have been seen by the physician himself/herself, depending on the disease activity and response to treatment. Patients with sufficient treatment plan are usually referred to the nurses at every second visits.

### 2.5 Diagnosis of RA

The 1987 American College of Rheumatology (ACR) revised criteria were applied to diagnosis of RA in the study population.^[20]^ Since 2010, diagnosis of RA was established according to the new 2010 ACR/ EULAR for RA.^[21]^

### 2.6 Data collection

Data including patients’ demographics, date, and types of visit, and also responsible physicians or nurses for all outpatient visits, planned by senior rheumatologists, junior physicians in rheumatology residency training, and specialist nurses (who have trained in the field of rheumatology), concerning patients with RA between November 2013 and November 2015, were extracted using Fyns Patient Administrative System (FPAS). These data for the mentioned period were chosen since it was the past 2 years FPAS was used to monitor patients’ visits. In FPAS, it was possible to see the initials of employees who treated the patient, patient's diagnosis, and type of consultation. The Danish registry of physicians was used to find respective physician graduation year and total years of experience as well. Exclusion criteria were the visits that were planned to pick up medicine or acute visits when intra-articular steroid injection was needed, since a conventional visit was not performed, leading to reduce bias. Visits for physicians, not in the rheumatology residency training, who worked for a short period of time, were also excluded from the study.

To monitor the quality of RA treatment, we used the DAS28 and HAQ scores, and measured the DAS28 and HAQ scores 3 times at following consultations: first at 1 of the physician consultations, second after nurse consultation, and finally at the third visit, either by a nurse or physician. These 3 HAQ/DAS28 scores were indicative of HAQ/DAS28 scores at 3 consecutive outpatient visits during a specific period of time, that is, November 2013 to 2015, and were not the same as HAQ/DAS28 scores measured at initial visits (first/second/third), when a newly diagnosed patient was referred to the outpatient clinic. The differences of DAS28 and HAQ scores at these consultations were calculated as well, which made it possible to determine the outcome of treatment when a consultation with a specialist nurse was performed. Data concerning DAS28 calculation (DAS28 components) and HAQ score were extracted/calculated from Danbio registry.

The average yearly salary of physicians and nurses was obtained from the section of economy at our department. The purpose of this was to get an idea of physicians’ salaries at different stages in their career, and also nurses’ salaries, which illustrate the approximate additional costs that should be borne by the department.

### 2.7 Variables and statistical analysis

The mean intervals between consultations for all senior and junior physicians working at the outpatient clinic during the mentioned period were calculated. This interval refers to the period of time between 2 consecutive consultations for each individual RA patient, which was planned by either physician, or at following visits by physician himself/herself or one of the specialist nurses working at the outpatient clinic. The nurse/physician visits ratio and physician efficiency index were calculated as mentioned below:

1. Nurse/physician visits ratio: Number of following visits by nurses divided by number of visits by an individual physician, which shows how often physicians referred RA patients to nurses during follow up visits.
2. Physician efficiency index: Nurse/physician visits ratio multiplied by the mean interval for an individual physician. The final result of physician efficiency index is indicator of both nurse/physician visits ratio and the mean interval.

Physician postgraduate experience (year) was considered as time interval (years) between graduation and the date that data were collected.

We additionally calculated delta DAS28 (ΔDAS28) twice as follows: firstly, ΔDAS28_1_ = DAS28 (first visit [by physicians]) − DAS28 (second visit [by nurses]), representing treatment outcome after physician visits; and second, ΔDAS28_2_ = DAS28 (second visit [by nurses]) − DAS28 (third visit [by physician or nurse]), representing treatment outcome after nurse visits to reveal patients outcome after nurse consultations. DAS28 was calculated using the following formula: DAS28 = 0.56∗√ (TJ) + 0.28∗√ (SJ) + 0.36∗ln(CRP + 1) + 0.014 × PGA + 0.96.

The Stanford Health Assessment Questionnaire was used to measure HAQ score. ΔHAQ_1_ = HAQ (first visit [by physicians]) − HAQ (second visit [by nurses]) and ΔHAQ_2_ = HAQ (second visit [by nurses]) − HAQ (third visit [by physician or nurse]) were measured in the same way.

Statistical analyses were performed using Microsoft Excel 2010. Continuous data were presented as mean ± standard deviation (±SD). Comparisons of the mentioned variables, between senior rheumatologists and junior physicians, were made by Student *t* test. To delineate the relationship between physician postgraduate experience and physician efficiency index, multiple linear regression analysis was performed considering the baseline disease activity and number of patients for each physician as potential confounders. The latter was done because of the fact that fewer senior rheumatologists were available in the outpatient clinic compared with junior physicians, provoking thought that the higher referral rate of nurse consultation was driven by heavier work load, but not the postgraduate experience of the physicians. *P* value was significant if *P* ≤0.05. Pearson correlation coefficient was used to measure the impact of postgraduate experience on the ratio of nurse consultation and physician efficiency index. In case of missing data, we used pair-wise deletion to keep as many cases as possible for each analysis.

## 3 Results

**Figure 1:**
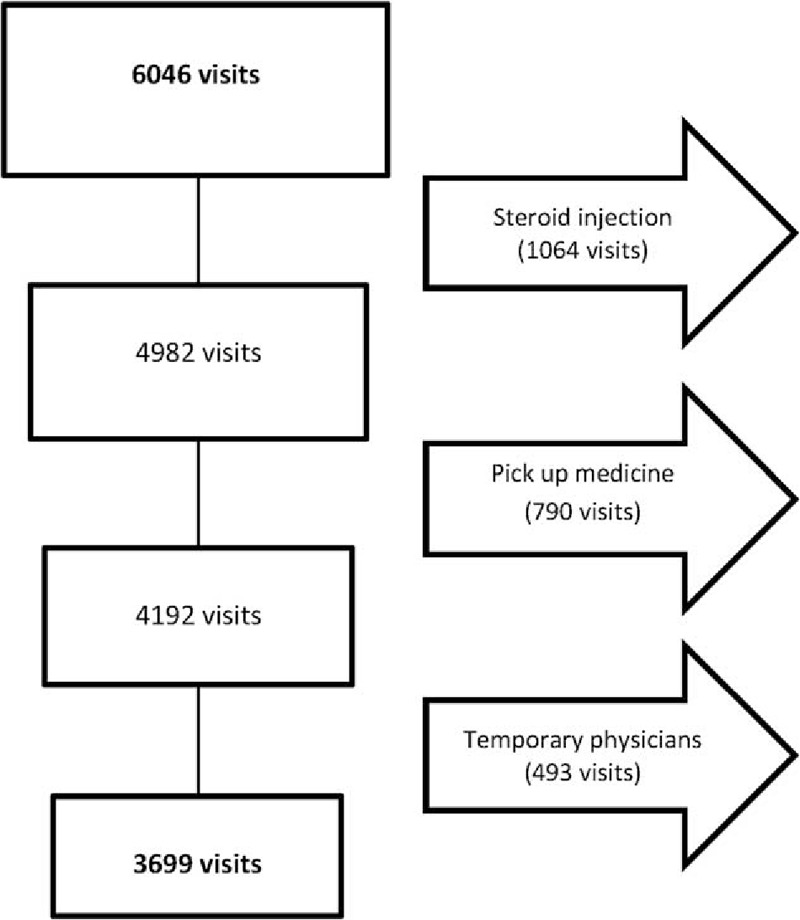
Study flow diagram, illustrating the included visits and reasons of exclusion.

Of 6046 visits, 3699 visits, planned by 11 physicians including 4 specialists in rheumatology and 7 junior physicians in rheumatology residency training, were included in this study. (Fig. 1) The numbers of visits belonged to 672 RA patients, of which 431 (64.1%) patients were female, the mean age being 64.9 ± 14.1 years, and DAS28 at baseline was 4.5 ± 1.2. There was a statistically significant difference between the nurse/physician visits ratios of senior rheumatologists and junior physicians (*P* = .01). Additionally, the mean efficiency index of senior rheumatologists was significantly higher than that of junior physicians (*P* = .04) (Table 1). Table 2 summarizes the nurse/physician visits ratios and physician efficiency indices according to the physician postgraduate experience.

**Table 1:**
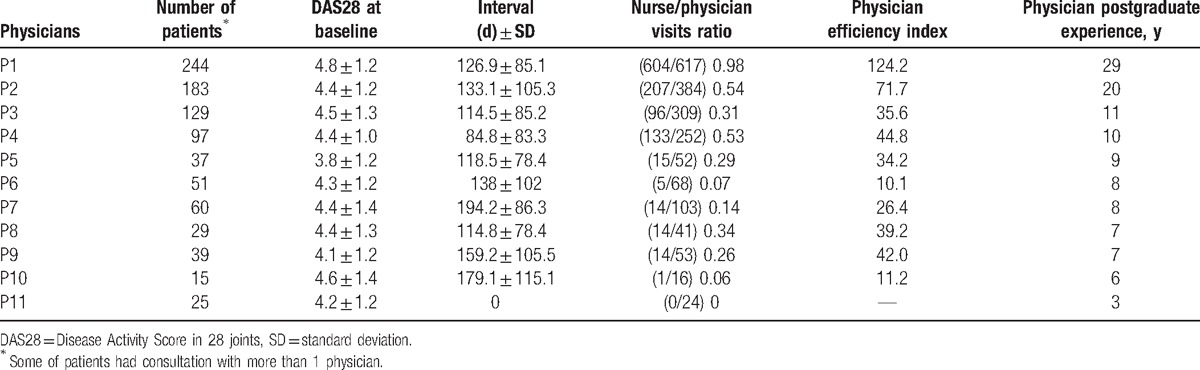
Number of patients for each physician, mean DAS28 at baseline, mean interval between consultations, nurse/physician visits ratio, physician efficiency index, and physician postgraduate experience of specialists in rheumatology (P1–P4, n = 4) and junior physicians in rheumatology residency training (P5–P11, n = 7).

**Table 2:**
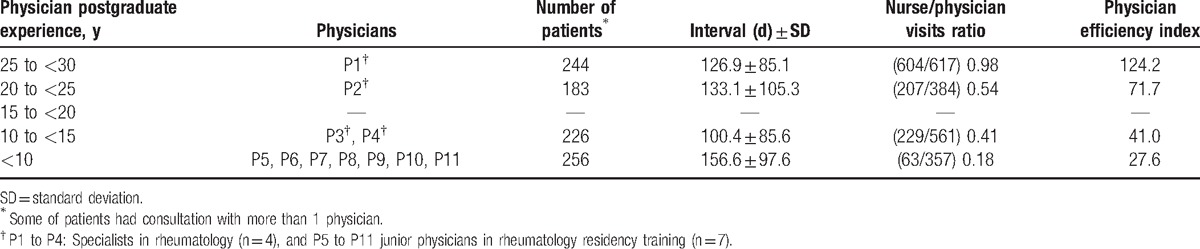
Number of patients for each physician, the mean interval between consultations, nurse/physician visits ratio, and physician efficiency index based on physician postgraduate experience.

Regression analysis illustrated a positive correlation between physician postgraduate experience and physician efficiency index adjusted for DAS28 at baseline and number of patients for each physician (regression coefficient 5.427, 95% confidence interval [CI] 1.068–9.787, *P* = .022) (Table 3). Given the small sample size (n = 11), we performed a post hoc power calculation on the basis of the following criteria: n = 11, adjusted *R*^2^ = 0.86, and 3 predictors, which revealed a power of 98% and 69% at a probability level of 0.01 and 0.001, respectively,^[22]^ although there are controversies regarding this type of calculation.

**Table 3:**
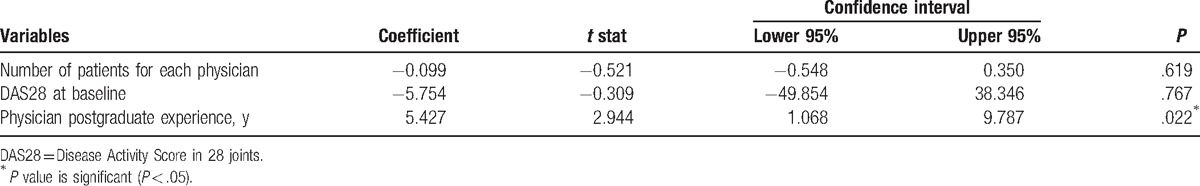
Result of multiple linear regression analysis for prediction of physician efficiency index.

**Figure 2:**
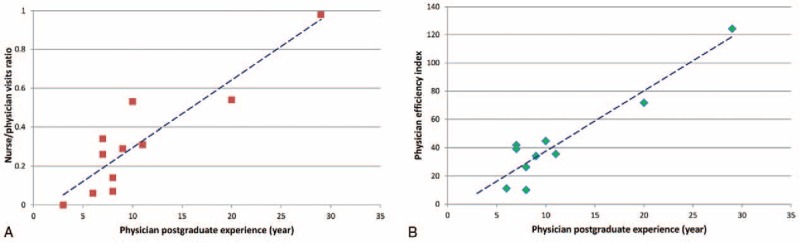
(A) High correlation between physicians’ postgraduate experience and the rate of nurse/physician visits ratio (*r* = 0.91). (B) High correlation between physicians’ postgraduate experience and physician efficiency index (*r* = 0.94).

Results of correlation analysis showed a significant high correlation between physicians’ postgraduate experience and the ratio of nurse/physician visits (*r* = 0.91) (*P* < .001) (Fig. 2A), and also physician efficiency index (*r* = 0.94) (*P* < .001) (Fig. 2B).

**Figure 3:**
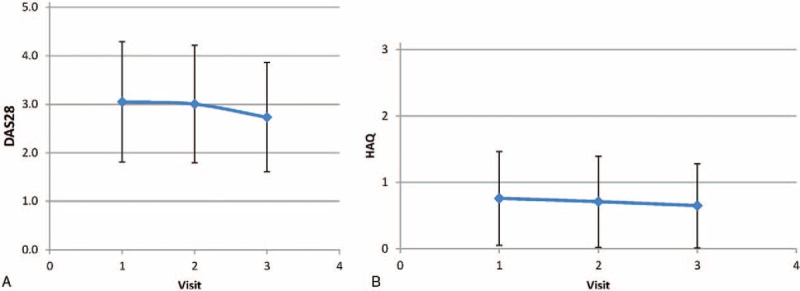
(A) Curve of the means of Disease Activity Score in 28 joints ± standard deviation at first (by physicians, 3.05 ± 1.24), second (by nurses, 3.01 ± 1.21), and third (by physician or nurse, 2.73 ± 1.13) visits. (B) Curve of the means of Health Assessment Questionnaire scores ± standard deviation at first (by physicians, 0.759 ± 0.707), second (by nurses, 0.709 ± 0.686), and third (by physician or nurse, 0.649 ± 0.634) visits.

There was a statistical difference between the mean of ΔDAS28_1_ and ΔDAS28_2_ (ΔDAS28_1_: −0.03 ± 1.17 and ΔDAS28_2_: 0.25 ± 1.01; *P* = .01). The means of ΔHAQ_1_ and ΔHAQ_2_ were 0.037 ± 0.364 and 0.032 ± 0.315, respectively (*P* = .86). DAS28 and HAQ scores were significantly decreased if physician visits were followed by nurse visits (*P* = .004 for DAS28 and *P* = .025 for HAQ) (Fig. 3A and 3B).

Yearly average of salary for physicians, at each stage of their career, and nurses, together with differences in salaries per year, has been summarized in Table 4.

**Table 4:**
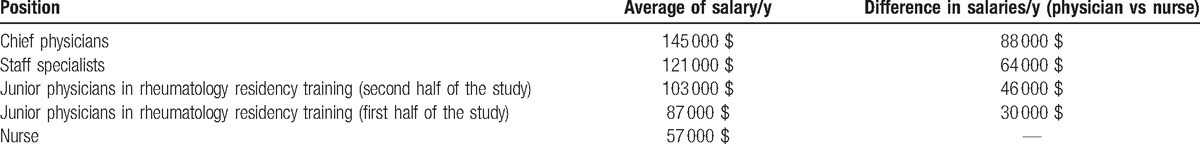
Approximate average of salary for physicians and nurses per year together with difference in salaries.

## 4 Discussion

This is the first cohort study, evaluating the physician efficiency regarding patients with RA. The key results of this study can be summarized as follows:

1. The tendency to plan an upcoming consultation with a nurse became higher as the experience of physicians increased. This will be a source of human resource waste and incur additional cost to the department, if junior physicians with less experience are reluctant to seek nurse consultations sufficiently. At our department, there is approximately a yearly difference of 30,000 to 46,000 American dollars per physician between nurse staffing and junior physicians’ income.
2. Senior rheumatologists had a significantly higher index of efficiency.
3. The total years of experience after graduation had predictive role for physician efficiency index after adjustment for DAS28 at baseline and number of patients for each physician.
4. The total years of experience after graduation were highly correlated to the ratio of nurse consultation sought by physicians and physician efficiency index.
5. Treatment outcome, evaluated by ΔDAS28/ΔHAQ, did not deteriorate when the patients were seen by nurse staffing.

The implementation of measures to assess the efficiency and value of healthcare system is critical and is the mainstay of efficiency appraisal. This leads to make a more efficient healthcare system and judicious use of resource, and brings advantages to patients and society as well. However, it is worth mentioning that costly care is not necessarily inefficient and misunderstanding of this and restraining costs without consideration of the consequences may deteriorate the condition and results in additional costs in future.^[3]^

Different measures were used to evaluate the efficiency of physicians in the previous literatures.^[2,23–27]^ Many of these measures relied on methods such as Data Envelopment Analysis and Stochastic Frontier Analysis, though ratio-based measures (eg, relative value units) were also common.^[2,27]^ The complexities of these methods, together with difficulties in interpreting results, have limited the use of them beyond clinical research area.^[2]^ Furthermore, these measures are mostly used in the United States with a different healthcare system, compared with Denmark, and are not necessarily generalizable to other countries. Apart from this, the reliability and/or validity of the measures used were still in doubt in many instances.^[27]^ The concept of physician efficiency index and nurse/physician visits ratio has been studied to some extent in the field of primary care with respect to reducing cost and maintaining the quality of care by substitution of general practitioners with nurses.^[28–32]^ The outcomes investigated varied widely across studies, for example, patient outcomes, process of care, resource utilization, and cost, depending on the study's focus. The results were, in general, purely descriptive and indicative of valuable contribution of nurses to reduce healthcare cost.

Based on the results of this study, we can conclude that physicians with more postgraduate experience work more efficiently. This is in line with earlier studies, illustrating the role of individual physician characteristic in efficiency. A study by Conrad et al^[26]^ revealed that sex, together with physicians’ experience, are significantly associated with productivity. Furthermore, postgraduate experience was a determinant of reduced resource use and healthcare costs.^[13–15]^ Our results were also in favor of the significant role of physician experience to improve physician efficiency, However, in the present study, we did not evaluate the possible relationship between physician characteristics (eg, age, sex, race/ethnicity, etc) and nurse/physicians visits ratio, and also physicians’ efficiency index, because we had data for only a few numbers of physicians (n = 11) who had worked at the outpatients clinic.

Another significant debate that may happen in this study is that nurse consultation may contribute to worsening of patient outcome; however, our results illustrated a statistically significant improvement in treatment outcome in these selected RA patients, after nurse consultation, which was assessed by comparing 3 consecutive DAS28 and HAQ scores. Our results did not compare treatment outcome in patients who were consulted with physicians and those who were seen by nurses; however, this could be examined in a prospective study with 2 well-defined groups of patients.

Our study had some strengths and limitations. The main strength of the study was the retrospective design. Neither of physicians/nurses, nor the authors knew that the study would be planned at the time of consultations, leading to minimization of the information bias as much as possible. An important issue that arises regarding the efficiency measurement, which is also a limitation of this study, is whether the results are comparable, because they are dependent on clinical characteristics of individual patient, health provider, geographic area, and so on. Another limitation of this study is that visits for only limited numbers of physicians in 1 single center were included in this study. Moreover, there was a lack of previous literature. No studies were found testing our hypothesis in a similar way. Our hypothesis in this study was made based on the expert opinion. The authors believe that the results of the current study are generalizable to other departments, because of broad and feasible inclusion criteria. However, further studies, with different patient populations involving multiple centers, should be performed to confirm our results.

In conclusion, this is the first study of its kind, to the best of our knowledge, which evaluated the physicians’ efficiency in a clinical setting. Junior physicians should be supervised to delegate responsibilities to nurses. They should learn to refer selected patients with milder disease and a well-proceeding treatment plan to nurses. Therefore, they will become more self-confident and their trust in nurses will increase as well. As a result, the entire department will operate more efficiently. Furthermore, nurses should be trained to evaluate patients for swollen and tender joints. Quality of care should be monitored continuously by indicators that are defined by Danish Danbio for instance markers of disease activity and results of CRP.^[16]^ At last, we think that our results merit detailed consideration by supervisors or organization managers who deal with decision-making in medical groups.

## Acknowledgments

We thank Mrs Maryam Mousavi and Mrs Merete Birkholm Hansen for their contributions to data collection.
